# Supplementary material for: Modeling of Large-Scale Functional Brain Networks Based on Structural Connectivity from DTI: Comparison with EEG Derived Phase Coupling Networks and Evaluation of Alternative Methods along the Modeling Path
Source: PLoS Comput Biol. 2016 Aug 9;12(8):e1005025. doi: 10.1371/journal.pcbi.1005025 (PMC4978387; doi:10.1371/journal.pcbi.1005025)
Supplement: S1 Text — Detailed description of empirical data recording procedures. (PDF) [file pcbi.1005025.s001.pdf]

S1 Text: Empirical Data

Participants

Seventeen healthy volunteers (7 women, mean age 65.6±10.9 std) underwent DTI and EEG resting-state recordings. None of the participants reported any history of serious medical, neurological or psychiatric diseases. None of the participants were taking any central nervous system-active medication. The study design was approved by the Local Ethical Committee of the Medical Association of Hamburg (PV 3777). All participants gave their written informed consent according to the ethical declaration of Helsinki.

MRI data acquisition

Structural imaging data were acquired using a 3 Tesla Siemens Skyra MRI scanner (Siemens, Erlangen, Germany) and a 32-channel head coil to acquire both diffusion-weighted and high-resolution T1-weighted anatomical images. For diffusion-weighted imaging, 75 axial slices were obtained covering the whole brain with gradients (b=1500 mm^2/s) applied along 64 non-collinear directions with the sequence parameters: Repetition (TR) = 10000 ms, echo time (TE) = 82 ms, field of view (FOV) = 256x204, slice thickness (ST) = 2 mm, in-plane resolution (IPR) = 2x2 mm. The complete dataset consisted of 2 x 64 b1500 images and additionally one b0 image at the beginning and one after the first 64 images. For anatomical imaging, a three-dimensional magnetization-prepared, rapid acquisition gradient-echo sequence (MPRAGE) was used with the following parameters: TR = 2500 ms, TE = 2.12 ms, FOV = 240x192 mm, 256 axial slices, ST = 0.94 mm, IPR = 0.94 x 0.94 mm.

DTI data preprocessing and cortical parcellation

Diffusion-weighted images were analysed using the FSL software package 5.1 (http://www.fmrib.ox.ac.uk/fsl). All datasets were corrected for eddy currents and head motion. Fractional anisotropy (FA) maps were calculated fitting the diffusion tensor model at each voxel. Structural T1-weighted anatomical images were processed using the Freesurfer software package 5.3.0 with standard procedures and parameters resulting in a cortical parcellation of 68 cortical regions [1, 2, 3]. Accuracy of cortical parcellation was checked visually. Two homologous regions (left and right entorhinal cortex) were discarded from further analysis due to frequent imaging artefacts surrounding this area of the brain. The remaining set of 66 parcellated brain regions were used for further analysis. Registration of structural and diffusion images was achieved using linear and non-linear transformation tools implemented in FSL [4]. Each cortical parcellation was transformed to diffusion space using the non-linear transformation coefficient file and accuracy of registration checked individually.

Fiber tractography and structural connectome construction

Processing of diffusion data included application of a probabilistic diffusion model, modified to allow estimation of multiple (n=2) fiber directions using the program bedpostx [5, 6]. From each seed ROI voxel, 10000 samples were

initiated through the probability distribution on principle fiber direction. Tracking resulted in individual maps representing the connectivity value between the seed ROI and individual voxels. Structural connectivity between two regions was measured masking each seed ROI results by each of the remaining ROI's. In probabilistic tractography, connectivity distribution drops with distance from the seed mask. We calculated the average length between different ROI's using the distance-correction option of probtrackx following recommendations from the online documentation of the FSL library. Values of average distances between seed and target ROI were applied alternatively to the reference method (Figure 1) to account for the confounding effect of tract length.

EEG data acquisition and analysis

Continuous EEG was recorded from 63 cephalic active surface electrodes arranged in the 10/10 system (actiCAP®, Brain Products GmbH, Gilching, Germany) during eight minutes eyes-open resting-state. Impedance was kept below 20 kΩ. Data were sampled at 1000 Hz, referenced to the Cz-electrode (actiCHamp® amplifier, Brain Products GmbH, Gilching). One electrode was mounted below the left eye for EOG-recording. Electrode positions were registered using an ultrasound localization system (CMS20, Zebris, Isny, Germany) before EEG-recording. Subjects were instructed to fixate a stationary fixation cross (viewangle $\pm 5°$) to reduce eye movements and were asked to avoid eye blinks, swallowing, any other movements and mental tasks like counting. The continuous EEG was offline rereferenced to a common cephalic average, demeaned, detrended and subjected to an independent component analysis (logistic infomax ICA; [7]) to remove eye-blink artifacts which were mostly reflected in 1-2 components. The data was downsampled to 125 Hz and segments containing artifacts like muscle activity, lead movements, electrode artifacts or incompletely rejected blink artifacts were removed visually.

The source activity was reconstructed using different inverse solutions:

1. an LCMV beamformer constrained by the covariance of the sensor data [8],
2. an ELORETA spatial filter [9] or
3. the MNE [10].

As a forward model we computed a boundary element method volume conduction model [11] based on individual T1-weighted structural MRI of the whole brain and individual electrode positions using the source space modeling functions of the SPM12 toolbox. The source time series were band pass filtered at the alpha frequency band (8 ± 2 Hz) and a Hilbert transform applied. From there functional connectivity estimates were derived as explained above. Analysis was performed with the FieldTrip package for MEG/EEG data analysis [12] and the Statistical Parametric Mapping software (SPM12b, Wellcome Trust Centre for Neuroimaging, London, UK, http://www.fil.ion.ucl.ac.uk/spm) on MATLAB Version 7.12.0 (R2011a, The Mathworks Inc., Massachusetts, USA).

References

[1] Dale AM, Fischl B, Sereno MI. Cortical surface-based analysis: I. Segmentation and surface reconstruction. Neuroimage. 1999;9(2):179–194.

[2] Fischl B, Dale AM. Measuring the thickness of the human cerebral cortex from magnetic resonance images. Proceedings of the National Academy of Sciences. 2000;97(20):11050–11055.

[3] Fischl B. FreeSurfer. Neuroimage. 2012;62(2):774–781.

[4] Jenkinson M, Beckmann CF, Behrens TE, Woolrich MW, Smith SM. Fsl. Neuroimage. 2012;62(2):782–790.

[5] Behrens T, Berg HJ, Jbabdi S, Rushworth M, Woolrich M. Probabilistic diffusion tractography with multiple fibre orientations: What can we gain? Neuroimage. 2007;34(1):144–155.

[6] Behrens T, Johansen-Berg H, Woolrich M, Smith S, Wheeler-Kingshott C, Boulby P, et al. Non-invasive mapping of connections between human thalamus and cortex using diffusion imaging. Nature neuroscience. 2003;6(7):750–757.

[7] Makeig S, Bell AJ, Jung TP, Sejnowski TJ, et al. Independent component analysis of electroencephalographic data. Advances in neural information processing systems. 1996;p. 145–151.

[8] Van Veen BD, Van Drongelen W, Yuchtman M, Suzuki A. Localization of brain electrical activity via linearly constrained minimum variance spatial filtering. Biomedical Engineering, IEEE Transactions on. 1997;44(9):867–880.

[9] Pascual-Marqui RD. Discrete, 3D distributed, linear imaging methods of electric neuronal activity. Part 1: exact, zero error localization. arXiv preprint arXiv:07103341. 2007;.

[10] Dale AM, Liu AK, Fischl BR, Buckner RL, Belliveau JW, Lewine JD, et al. Dynamic statistical parametric mapping: combining fMRI and MEG for high-resolution imaging of cortical activity. Neuron. 2000;26(1):55–67.

[11] Oostenveld R, Stegeman DF, Praamstra P, van Oosterom A. Brain symmetry and topographic analysis of lateralized event-related potentials. Clinical neurophysiology. 2003;114(7):1194–1202.

[12] Oostenveld R, Fries P, Maris E, Schoffelen JM. FieldTrip: open source software for advanced analysis of MEG, EEG, and invasive electrophysiological data. Computational intelligence and neuroscience. 2010;2011.
