# Supplementary figures and images for: Modeling of Large-Scale Functional Brain Networks Based on Structural Connectivity from DTI: Comparison with EEG Derived Phase Coupling Networks and Evaluation of Alternative Methods along the Modeling Path
Source: PLoS Comput Biol. 2016 Aug 9;12(8):e1005025. doi: 10.1371/journal.pcbi.1005025 (PMC4978387; doi:10.1371/journal.pcbi.1005025)

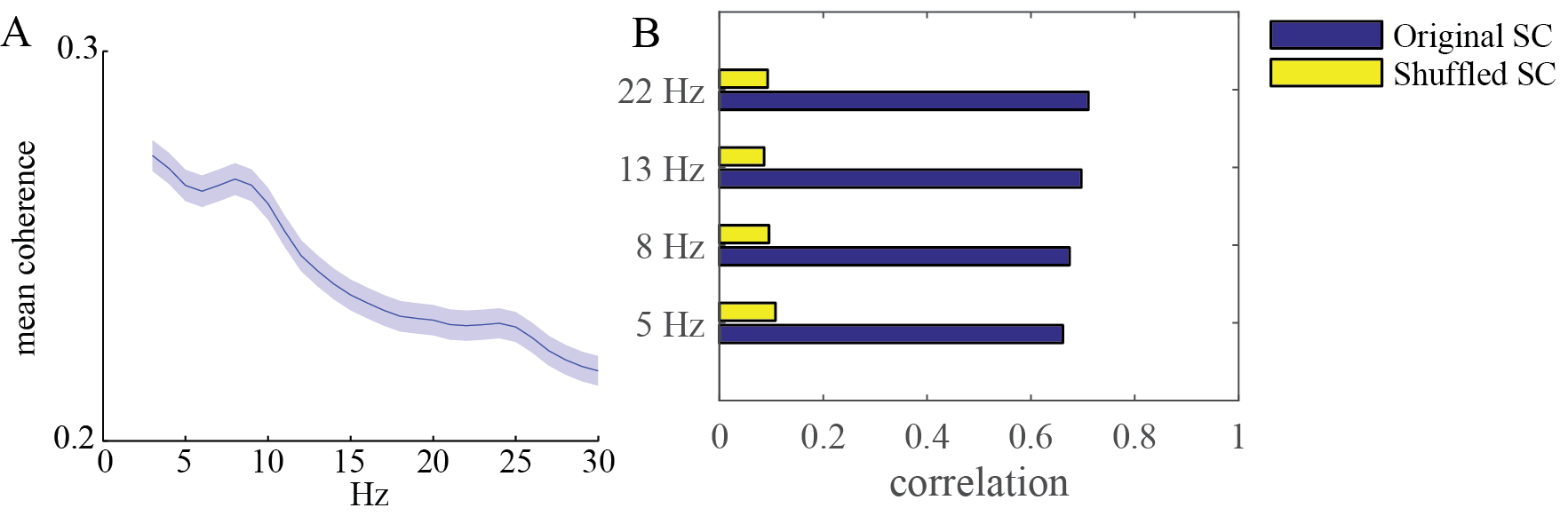

Supplement: S1 Fig — A: The mean coherence values (±SEM, shaded area) between all ROIs (n = 2145) is calculated for the frequency range of 3–30 Hz. Overall coherence at lower frequencies is higher with a peak around 8 Hz and a smaller peak around 24 Hz. B: The model performance at different bandpass filters of the EEG source time series. (TIF) [file pcbi.1005025.s002.tif]

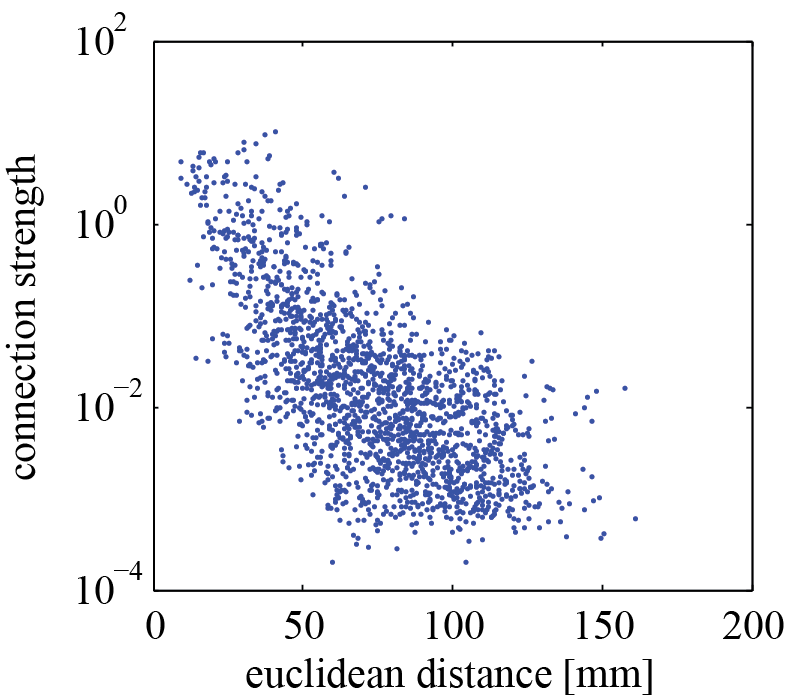

Supplement: S2 Fig — The euclidean distance is measured between the center coordinates of individual ROIs. The strength between ROIs are the number of tracked DTI fibers divided by the product of both ROI sizes. The logarithm of the structural connection strength is inversely correlated with the euclidean distance (r = −0.37, n = 1883, p < .0001). Connections with zero strength (pairs of ROIs with no probabilistic tracked fibers between them) were excluded (n = 262) due to the logarithmic axis. (TIF) [file pcbi.1005025.s003.tif]
